# Wireless Electrochemical Detection on a Microfluidic Compact Disc (CD) and Evaluation of Redox-Amplification during Flow

**DOI:** 10.3390/mi10010031

**Published:** 2019-01-07

**Authors:** Maria Bauer, Jaume Bartoli, Sergio O. Martinez-Chapa, Marc Madou

**Affiliations:** 1 Department of Mechanical and Aerospace Engineering, University of California Irvine, Irvine, CA 92697, USA; mebauer@uci.edu; 2 Department of Biomedical Engineering, University of California Irvine, Irvine, CA 92697, USA; jbartoli@uci.edu; 3 School of Engineering and Sciences, Tecnologico de Monterrey, Monterrey 64849, NL, Mexico; smart@tec.mx

**Keywords:** redox-amplification, CD microfluidics, wireless sensing, bioassay platform, collection efficiency, limit of detection, noise mitigation

## Abstract

Novel biomarkers and lower limits of detection enable improved diagnostics. In this paper we analyze the influence of flow on the lower limit of electrochemical detection on a microfluidic Compact Disc (CD). Implementing wireless transfer of data reduces noise during measurements and allows for real time sensing, demonstrated with the ferri-ferroyanide redox-couple in single and dual mode cyclic voltammetry. The impact of flow on redox-amplification and electrode integration for the lowest limit of detection are discussed.

## 1. Introduction

Out of the 19,613 human genes only about 15% have one or more secreted proteins, thus making up a total of 2933 theoretically detectable secreted proteins [1]. These proteins comprise a potential source of biomarkers for various states of cells in real time [2]. However, Food and Drug Administration (FDA) approval has only been obtained for the diagnosis of 149 of those proteins [1,3]. The reason for the lack of diagnostics covering further potential biomarkers and thus enhancing the field of diagnosable diseases and improving health conditions is often the necessary but unreached limit of detection (LOD) of the diagnostic to reliably detect very low concentrations of proteins [4,5]. This need fuels the development of improved diagnostic test systems and detection methods.

Microfluidic systems often excel in improving LOD and reproducibility, while only requiring very small amounts of sample and reagents [6]. Furthermore, they allow for fully automated bioassays, enabling Point of Care (POC) diagnostic devices, which integrate functions of otherwise expensive lab equipment (e.g., lysis, incubator, microscope, centrifuge, etc.) on one fully automated platform. Reduced in size and without the need for technical personnel to conduct many steps on separate machines, detection times are shortened [7]. One microfluidic platform which excels in its simplicity for pumping, automation of many assay steps, air-bubble free fluidics and multiplexing is the microfluidic disc, also known as microfluidic Compact Disc (CD) [8]. Fluids here are driven by the centrifugal force provided by the rotation of a disposable disc, while the contamination risk is reduced as compared to conventional multi-step analysis through the elimination of intermediate transport from device to device via interfaces to the outside world [9].

Various detection methods have been employed on CD microfluidic platforms, usually based on optical, non-contact techniques, such as absorbance, fluorescence and chemiluminescence [8,10]. However, due to the proportionality of absorbance to optical path length most commercial absorbance-based systems are based on rather thick optical cells (equal or larger than 10 mm) and not suitable for miniaturization [8]. Fluorescence and luminescence-based systems afford very good LODs but at the cost of complexity and size of the instrumentation [10]. Generally, optical detection methods also require the stopping of the disc for readout and often even transportation of the disc to a different read-out device [8].

Unlike absorbance-based systems, electrochemical detection benefits from miniaturization through enhanced mass transport leading to improved LOD and shorter response times [8,10,11]. Additionally, electrochemical detection is feasible in turbid solutions and is independent from an optical path length. Without the need for optical grade materials or peripheral equipment with high electrical power demands, electrochemical detection affords for smaller, portable and lower cost diagnostic devices [8,11].

### 1.1. Current Methods for Interfacing Electrochemical Detection on a Microfluidic CD

Similar to optical detection, electrochemical detection often requires stopping of the disc for measurement in order to ensure electrical connectivity between the electrochemical sensors on the CD and a stationary instrument [12,13]. This spin-stop solution results in undesirable delays for time-sensitive experiments and cannot take advantage from the signal enhancement expected due to flow. An electrochemical signal is indeed enhanced through enhanced mass transport increasing the amounts of analyte reaching the electrode surface. When mitigating unwanted migration via increased ionic concentration in the solution, only convection aids the otherwise purely diffusion-based mass transport of analyte to the electrodes. Increasing flow and thus convection hence leads to higher current readouts and therefore lowers LOD and increases sensitivity [14].

In order to take advantage of flow over the electrodes, avoid delays or altered signal readouts, measurements thus need to be taken while the disc is spinning. This has been realized before through the use of a so-called slip-ring setup, allowing stationary contact pads to conduct electricity to and from a set of rotating rings spinning along with the rotating CD [14]. However, the physical contact induces significant electrical noise while wearing out components and thus gradually changing conductivity and resistance. An alternative has been demonstrated using a mercury based rotating electrical connector, inducing lower amounts of noise in the measurements during spinning of the disc [15]. However, this connection method is made up of a rather bulky setup and relies on rather expensive and undesirable mercury as the conducting and connecting substance. Rather than establishing electrical contact with the sensors on the disc, recently efforts have been made to develop wireless power and data connections. The first prototypes employing this approach demonstrate that the elimination of the physical interface reduces the electrical noise and increases the life-time of the device [16]. 

In this work, we aim to add to the relatively few examples of indirect interfacing methodologies by presenting a setup that integrates the analysis instrument, a bipotentiostat (allowing for simultaneous sensing of two working electrodes), to the spinning platform, powering it with a small battery and sending the obtained data via Bluetooth to a stationary desktop computer or mobile phone. Thus, rather than conducting the very low current signals to a stationary instrument, the obtained current signal is sent wirelessly as a binary code and is not affected by transmission errors and noise due to changing electrical contacts.

### 1.2. Redox Cycling Amplification using Interdigitated Microelectrode Arrays

While most the efforts made in the field of electrochemical detection on a microfluidic CD have relied on the use of microelectrodes in a 3 electrode setup with a working electrode, reference electrode and a counter electrode—so called single mode [12,13,14], here we use a 4 electrode setup utilizing 2 working electrodes besides a reference and a counter electrode—called dual mode. The working electrodes consist of sets of interdigitated electrode fingers to further enhance the performance of the electrochemical detection. This type of electrode configuration is called an Interdigitated Electrode Array (IDA)). IDAs allow for redox amplification (RA) of a measured signal by the redox-cycling of a species between the interdigitated digits, in which a redox species is transported multiple times between the two sets of working electrodes before diffusing out into the bulk of the test solution. This amplification results in higher currents for a given concentration of analyte and therefore is capable of lowering the LOD [17]. The materials’ choices and manufacturing means for IDAs vary and severely impact electrode cost, which especially for disposable systems is a major consideration.

Generally, RA has been demonstrated to significantly enhance the current signal and to decrease the LOD through an increase in sensitivity. Odijik et al., for example, demonstrated an RA of over 2000 using a parallel plate setup with ferrocyanide as the redox-couple and Dam et al. showed an RA of 60–70 with a high aspect ratio platinum IDAs with a width- gap- height of 2 μm- 2 μm- 7 μm [18,19]. Even though very high RA factors might allow for the detection of very low concentrations and might be applicable to very small sample volumes, the question arises whether such measurements have actual analytical value as they do not relate the measured currents back to a bulk concentration of sample not between the electrodes as required by Faraday’s law. Working with a cell allowing for flow over the electrodes during the measurement allows for the supply of new species from the bulk of the solution thus providing for a fast coupling of transport between the fingers of the electrode sets and the bulk.

The effect of flow on the current signals in 3 and 4 electrode setups was described previously [14,20]. While the analytical current in a single mode setup benefits from flow enhanced mass transport, leading to a 7 fold increase under flow (500 nL/s) compared to the no flow condition as demonstrated a study conducted by Kamath [20], dual mode currents show little enhancement under increasing flow. This phenomenon is explained by shearing off of the redox-species concentration profile through flow and therefore always hindering of the redox-species diffusing against the direction of the flow of reaching the other working electrode [17]. Kamath et al. describe a drop in RA from 37 to 4 on carbon IDAs under flow (500 nL/s) when compared to no flow [11]. Higher aspect ratio electrodes suffered less from the decrease in RA during flow which is explained by the lesser effect of the flow onto the redox-path of analyte between the digits of the electrodes rather than above them [20]. Besides RA, also the collection efficiency [CE = Collector Current (one of the working electrodes)/Generator Current (the other working electrode)] can be used as a measure for characterization of redox-cycling in dual mode.

Despite ample research in all three areas: diagnostics on a CD platform, flow enhanced electrochemical sensing and redox-amplification with IDAs, to our knowledge, no group has characterized the performance of IDAs in dual mode under flow conditions on a microfluidic CD nor have such measurements been transmitted wirelessly off the rotating platform.

## 2. Materials and Methods 

### 2.1. Microfluidic Disc Design and Fabrication

**Figure 1:**
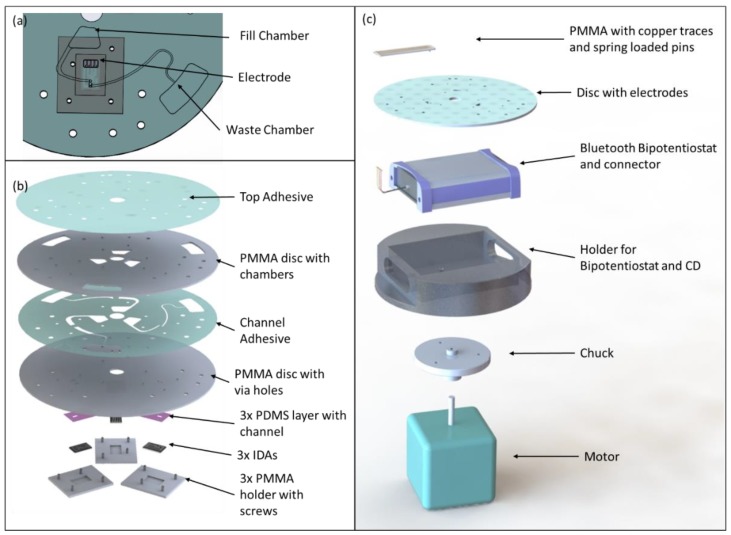
(**a**) Fluidics on Compact Disc (CD) and positioning of electrode underneath disc, (**b**) Assembly of modular CD. Electrodes can be removed for cleaning and replacement, (**c**) Test setup for non-contact measurements.

Platinum IDAs consisting of 2 working electrodes, counter, and reference electrode and with digit widths and gaps of 3 μm and electrode height of 90 nm were purchased from CH Instruments Inc. (Austin, TX, USA) and integrated onto the CD. Carbon IDAs were obtained through photolithography of SU8 and subsequent pyrolysis as described elsewhere [20]. The dimensions of the carbon IDA were identical to those of the platinum IDAs but with a height of 1.72 μm and 70 digits per working electrode compared to 65 pairs for the platinum IDAs. A drop of Ag/AgCl was used on top of the platinum and carbon electrodes as pseudo reference electrodes. The microfluidic disc and IDAs were assembled such, that the electrodes could be removed for cleaning (see Figure 1), which was essential for repeated usage of electrodes and discs for different and/or repeated experiments. The disc assembly consists (top to bottom) of a top adhesive layer sealing the chambers in an underlying polymethylmethacrylate (PMMA) disc, followed by another adhesive layer with microfluidic channels and another PMMA layer with through holes to provide flow to the electrodes. Between the latter PMMA layer and the electrodes a polydimethylsiloxane (PDMS) layer was placed with a channel perpendicular to the direction of the electrode digits and connecting the through holes while also exposing reference and counter electrodes. Finally, each electrode is held by a PMMA holder which is screwed to the microfluidic disc. Solutions were loaded into fill chambers and flown via the through hole into PDMS channels and onto the sensing area of the working electrodes. After returning via the other through hole in the disc fluid was collected in a waste chamber (see Figure 1).

### 2.2. Setup for Rotating Electrochemical Measurements

A spinstand with motor (PMB21B-00114-00, Pacific Scientific, Co., Duarte, CA, USA) and controller (PC3403Ad-001-E, Pacific Scientific, Co., Duarte, CA, USA) was used for rotary actuation, while a trigger (D10DPFPQ, Banner Engineering, Corp., Minneapolis, MN, USA) was installed to trigger a high-speed camera (Area Scan A311fc, Basler AG, Ahrensburg, Germany) and a stroboscope (DT-311A, Nidec-Shimpo, Corp., Kyoto, Japan) at each single rotation of the disc for visualization of the fluidics. This setup was used for the analysis of flow rates as well as for general control of fluidics (such as fill levels of the chambers).

A portable, battery powered bipotentiostat (μStat400, Metrohm DropSens AG, Herisau, Switzerland) was purchased and controlled with the Dropiew 8400 software (Metrohm DropSens AG, Herisau, Switzerland). To allow for integration of this instrument onto the spinning platform a holder was machined in which the bipotentiostat was placed and on top of which microfluidic discs could be mounted (see Figure 1c). The original DropSens cable was soldered to a connector consisting of a polymethylmethacrylate (PMMA) base with copper foil paths and spring-loaded gold pins for contacting the electrode bonding pads. Sensed data was sent via Bluetooth to a stationary computer for further analysis.

### 2.3. Procedures for Electrochemical Measurements

Solutions were prepared with 1 mM, 3 mM, 5 mM, 7 mM and 9 mM ferricyanide, ferrocyanide redox-couple and a supporting electrolyte of 2 M KCl. One solution was prepared as blank (2 M KCl) for measuring noise from the setup.

#### 2.3.1. Platinum IDAs

For single mode operation, the potential was scanned from 0 V to 0.6 V in 0.002 V steps at a scan rate of 0.02 V/s. Dual mode was conducted using the same step size and scan rate with one working electrode scanned from 0.1 V to 0.6 V and the second working electrode kept constant at 0 V. This scan rate leads to peak currents in no-flow and low RPM conditions due to the diffusion-limited mass transfer, while allowing for reaction limited currents at higher flow rates. Choosing a lower scan rate was not feasible as it did not allow for the completion of forward and backward scan with a maximum fill volume of 300 μL.

Platinum electrodes were cleaned, once degradation in performance was observed. Cleaning of the Pt electrodes consisted of depositing a drop of 0.1 N sulfuric acid solution on the sensing area of the Pt electrodes and conducting 20 cycles of cyclic voltammetry (CV) with sweep range from 0 V to 1.5 V at a scan rate of 0.1 V/s. This procedure was followed by rinsing the IDA with distilled water and drying with filtered nitrogen. When subsequent control under microscope showed debris between the digits of the IDA, the interdigitated electrodes were carefully polished parallel to the digit direction with a gloved finger under flowing distilled water. Drying and control with a microscope was repeated until all the debris was removed.

#### 2.3.2. Carbon Electrodes

**Figure 2:**
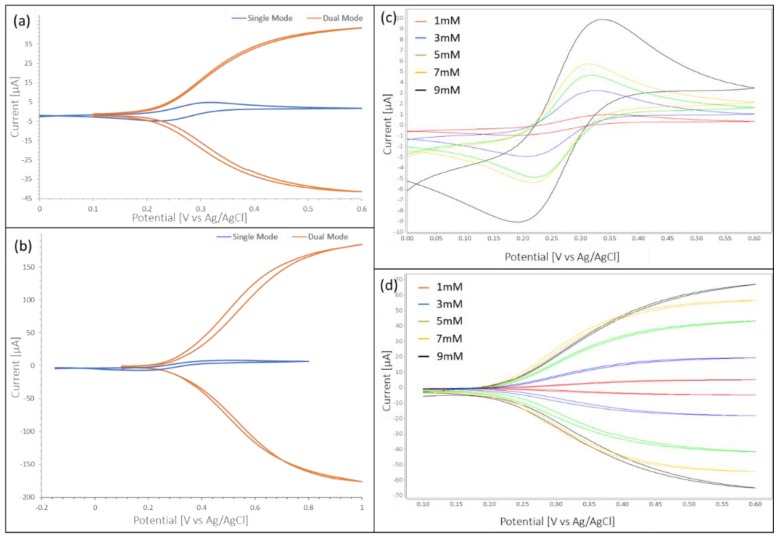
Single and dual mode cyclic voltammetry (CV) for (**a**) Platinum Interdigitated Electrode Array (IDA) and (**b**) carbon IDA, (**c**) Single mode CV for platinum IDA with concentrations 1 mM through 9 mM ferricyanide, ferrocyanide redox-couple, (**d**) Dual mode CV for platinum IDA with concentrations 1 mM through 9 mM ferricyanide, ferrocyanide redox-couple.

Similar to platinum electrodes, carbon electrodes were utilized in single as well as dual mode. For single mode the best scan range was 0 V to 0.5 V. The scan rate in single as well as dual mode was 0.01 V/s. Due to the lower conductivity of carbon as compared to platinum, the iR drop of carbon electrodes was higher. As the portable bipotentiostat did not allow for iR compensation, larger scan ranges were necessary to take full advantage of redox-amplification when working with carbon electrodes. The dual mode curves for carbon electrodes were observed to approach a plateau around 1 V (see Figure 2b) when scanning at 0.01 V/s. However, such large scan ranges lead to the disintegration of the counter electrode and therefore to the reduction of the lifetime of the carbon electrode. To allow for the maximum sweep range and avoid early disintegration of the counter electrodes, the pH level of the solutions was measured and adjusted to a pH of 8 using sodium hydroxide. Nevertheless, to allow for an increased lifespan of the electrodes, sweep ranges in dual mode were limited to 0.1 V to 0.6 V for experiments on the CD. Due to the lower cost (about $5 per carbon electrode versus about $200 for a platinum electrode), carbon electrodes were replaced once signals started diverging rather than cleaned and used again. Due to the high iR drop and the lower stability of carbon IDAs in the required potential range, tests were limited to stationary testing with a single concentration (5 mM). Taking all measurements (single and dual mode) on a single electrode was important as the PDMS channel, acting as shield in on disc measurements was manually positioned to only allow sensing area, counter and reference electrodes to be exposed. Small changes in positioning as well as tightening of screws lead to changes of areas exposed to fluid and therefore to changes in currents. 

### 2.4. Data Analysis

Single mode limiting currents were calculated with respect to the flow rate *ν* according to [21]:

(1) $$i_{L}=1.47nFC{\left(DA/B\right)}^{\frac{2}{3}}\nu^{\frac{1}{3}}$$

where *n* is the number of electrons transferred, *F* is Faraday’s constant, *C* is the bulk concentration, *D* is the diffusion constant, *A* the area of electrodes and *B* the height of the channel. Therefore, flow is expected to increase with current as the cubic root until the reaction rate at the electrode surface becomes limiting.

Dual Mode current without flow for different concentrations is calculated according to Aoki et al. [22]:

(2) $$i=mbnFCD\left(0.637\ln\left(\frac{2.55}{x}\right)-0.19x^{2}\right)$$

With *m* the number of digits per working electrode, *b* the length of each digit and *x* the width of the digit divided by the width plus the gap. The diffusion constant of potassium ferri-ferrocyanide was assumed at 7.26 × 10^−6^ cm^2^/s [23]. RA and CE in dual mode under flow are expected to decrease with increasing flow rates due to shearing off of species [20,24]. Dual mode currents with flow at different concentrations were calculated according to the model by Morita et al. [24].

Current voltage measurements (CVs) were obtained using Dropview 8400 software and analyzed with respect to the baseline of the respective curves. Such a baseline for each CV was established using a first order tangent based on 5 points in the linear area of each curve. RA and CE were calculated with RA = Limiting Current in Dual Mode/Limiting Current in Single Mode and CE with: CE = Collector Current/Generator Current. A calibration curve was established for each spin speed based on measurements of varying concentrations (i.e., 1 mM, 3 mM, 5 mM, 7 mM, 9 mM at spin speeds of 0 RPM, 100 RPM, 200 RPM, 300 RPM, 400 RPM respectively). The curves were quasi linear in the range of the tested solutions and the obtained slope equals the sensitivity at the respective spin speed. The LOD was calculated as 3 times the standard deviation of the CV for the blank solution in a range of 0.5 V around the potential of the peak currents in solutions with the redox couple present. The standard deviation as well as the noise levels were measured using Origin 2018b (Originlab Corp., Northampton, MA, USA) Oscillatory noise from spinning of the disc in the measured signal was removed utilizing Fast Fourier Transformation (FFT) Filtering.

## 3. Results and Discussion

### 3.1. Stationary Electrochemical Sensor Performance

First, stationary sensor performance was established in single and dual mode with a drop of 5 mM ferri-ferrocyanide solution on platinum as well as on carbon IDAs. Potentials were measured versus Ag/AgCl. In Figure 2 we show the resulting CV curves for platinum (a) and carbon (b) electrodes. Measurements for both electrodes are summarized in Table 1. While with platinum IDAs the area of the working electrodes exposed to fluid is restricted to the interdigitated digits by a passivation layer added by the manufacturer, for carbon IDAs in the droplet experiment a small area of the traces between digits and bonding pads to the solution may be exposed to the solution as well. This adds to generally higher currents with carbon electrodes for droplet measurements as well as a CE of slightly larger 1 (due to unequal areas of the two working electrodes exposed to solution). It can be seen from Table 1 that carbon IDEAs have a higher RA than platinum IDAs. This can be explained by the higher aspect ratio of the carbon IDAs compared to platinum IDAs. The higher aspect ratio of the carbon electrodes does not only increase the surface area for electron exchange but also decreases the diffusion path in a linear diffusion profile between electrode digits as compared to an elliptical diffusion profile atop of the electrode digits in the more planar Pt case[11]. In Table 1 we summarize the measurements depicted in Figure 2. It might be noted here that with different carbon electrode geometries carbon IDAs amplification factors of up to 38 were obtained. Predicted dual mode current (based on Equation (2)) for platinum IDAs is in good agreement with experiment, with calculated and experimental value respectively of 45.1 μA and 37.7 μA. This deviation seems to be largely within the variation when changing the setup (disassembly for cleaning of the electrode and subsequent reassembly) of the experiment. Additional possible reason for lower than calculated values in the experiments is described in the literature through contamination of the electrode surface [25].

In Figure 2c,d we show the CVs for platinum IDAs in stationary on disc measurements for various concentrations in single and dual mode respectively. For the dual mode graph, baselines were subtracted from measurements for better visualization of the current increase at higher concentration. 

**Figure 3:**
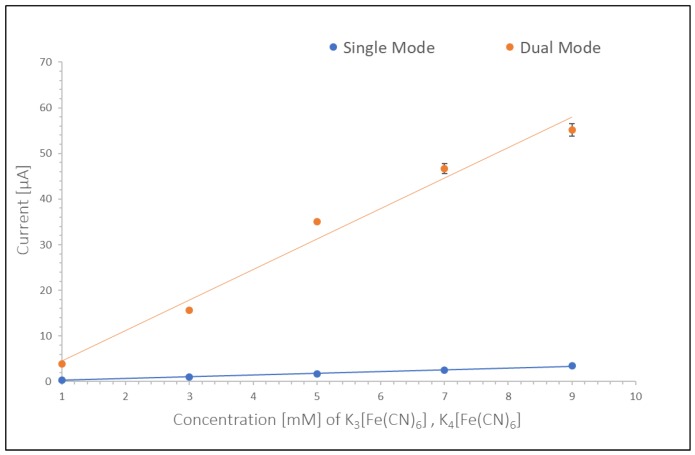
Calibration curve for single and dual mode, platinum IDA, stationary measurements.

Based on the measurements shown in Figure 2 calibration curves for single and dual mode of platinum electrodes were obtained. These are shown in Figure 3. The sensitivity in Figure 3 was calculated from the slope of calibration curve as 0.39 μA/mM and 6.7 μA/mM for single and dual mode in stationary measurements respectively.

As can be seen from these values and Figure 3, the sensitivity for dual mode is higher than for single mode, thus affording lower LOD of 10.14 nM in dual mode versus 63.51 nM in single mode at 0 RPM (based on average noise level of 21.5 pA in measurement). Current measurements varied around 10% between setups which could be influenced by the manual alignment of the channel on the sensing area as well as manual adjustment of clamping pressure onto the silicone layer (leading to changes in width and height of the channel). Measurements within one setup showed only small standard deviations of less than 1% as can be seen in the error bars in Figure 3.

### 3.2. Effect of Flow Velocity on Redox Cycling

**Figure 4:**
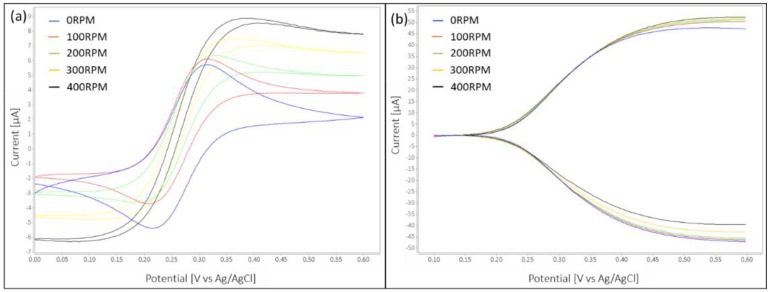
(**a**) Single mode platinum IDA CV for increasing flowrates. The behavior changes from diffusion limited to reaction limited for higher flow rate, (**b**) Dual mode CV for platinum IDA for different flowrates. The baseline was subtracted from curves in dual mode for better visibility of the current increase with increasing flowrate.

The effect of flow on the measured currents in single and dual mode can be seen in Figure 4a,b respectively. The graphs show the CV curves for 9 mM ferri-ferrocyanide solution. It is observed from Figure 4a that single mode CV moves from a diffusion limited curve with peak current to a reaction limited curve for increasing flow rate. The dual mode curves Figure 4b show less impact of flow on current but the current still increases with increasing flow.

**Figure 5:**
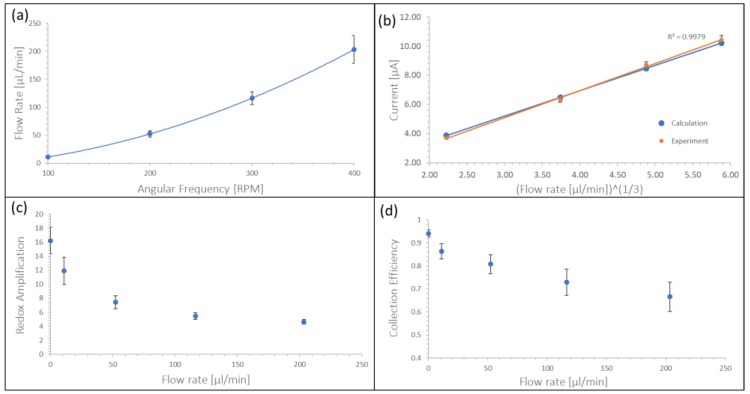
(**a**) Relation between angular frequency and flow rate, (**b**) Calculated (blue) and experimental Single Mode currents for increasing flow rate (platinum IDA) (**c**) Measured RA versus flow rate (Platinum IDA), (**d**) Measured CE versus flow rate (platinum IDA).

To allow for the calculation of single mode currents for different spin speeds, the flow rate at different spin frequencies was measured and results are shown in Figure 5a. Calculation (based on Equation (2)) and experimental single mode currents were in very good agreement (Figure 5b) and linear with $\nu^{\frac{1}{3}}$. In Figure 5c we show the decrease of RA with respect to the limiting current from 16.2 to 4.6 and Figure 5d shows the decrease in CE from 0.94 to 0.67 both with increasing flow rate. The RA and CE values are based on average measurements of all concentrations at the respective flow rates. This behavior is as expected as the increasing flow shears off redox species and precludes it from repeated redox cycling. Error bars in Figure 5 show standard deviations. In Figure 5b error bars represent standard deviation of different measurements within the same setup.

### 3.3. Flow-Enhanced Electrochemical Sensor Performance

Despite a decreasing RA, the dual mode with flow affords the highest sensitivity of 6.99 μA/mM and in conjunction with average constant noise level (after FFT filtering of oscillatory noise from the rotation) of 21.5 pA leads to lowest LOD of 9.23 nM. Therefore, based on this study, dual mode CV with flow should be used for best electrochemical measurement performance at low concentrations (see Table 2). This is because even at higher than tested flowrates, dual mode limiting current will only eventually approach single mode limiting current but cannot result in lower currents. This is because once redox-cycling is completely mitigated by flow, generator current will simply become single mode current. In CV measurements with very high scan rate, peak currents during stationary or slow flow measurements will increase and could lead to higher currents than the limiting current in dual mode. Fast CV experiments are however outside the scope of this work.

Table 3 shows a comparison of experimental and predicted dual mode currents with flow at a spin speed of 400 RPM. Overall experimentally obtained currents are by a factor of 4.3 to 5.6 higher than predicted currents, which could be due to smaller than assumed channel geometry (height and width of channel depends on compression and thus deformation of PDMS layer) as well due to neglecting of as species trapped between the digits at the height of the electrodes, which are less affected by flow.

## 4. Conclusions

In this work we studied the effect of flow on redox-amplification of platinum Interdigitated Electrode Arrays on a microfluidic disc and compared stationary currents obtained from carbon and platinum electrodes. A modular disc assembly allowed for cleaning and replacement of the electrodes. Cyclic Voltammetry measurements were conducted with a spinning bipotentiostat, placed underneath the fluidic disc, which was battery powered and transferred data via Bluetooth to a stationary computer, thus eliminating the need for physical contact to conduct data from the spinning electrodes to a stationary analysis device and therefore decreasing noise during measurements. Stationary cyclic voltammetry measurements were conducted on both, carbon and platinum Interdigitated Electrode Arrays, with carbon electrodes leading to higher currents and redox-amplification, while being more affordable than platinum electrodes. However, platinum electrodes showed to be more stable throughout experiments and had less iR drop than carbon electrodes, therefore reaching steady state currents faster. Calibration curves for platinum electrodes in stationary measurements showed a higher sensitivity at 6.36 μA/mM for dual mode as compared to 1.02 μA/mM in single mode, leading to a lower limit of detection of 10.14 nM in dual mode versus 63.51 nM in single mode.

After imposing flow over the electrode surface and increasing flow rate to 5.88 μL/min, a more than two-fold increase in sensitivity for single mode was observed while dual mode sensitivity increased less than 10% and redox-amplification and collection efficiency decreased. Lowest limit of detection of 9.23 nM was nevertheless obtained in dual mode at the highest tested flowrate. When targeting high sensitivity measurements this setup should therefore be chosen.

Future research should aim at integration of the bipotentiostat in a smaller and more robust format to improve spin performance. The currently utilized bipotentiostat weighs about 0.48 kg, significantly affecting inertia of the spinning modules and therefore negatively affecting oscillation and acceleration capability. Additionally, improved connection between bipotentiostat and electrodes could obviate the need for fast Fourier transform filtering of measurements. Finally, for application of electrochemical sensing to detect various biomarkers (e.g., secreted proteins), combination with ELISA (enzyme-linked immunosorbent assay) technique is a possible solution to enable indirect detection of non-redox active species. Through specific linkage of biomarkers to capture and detection probes and utilization of e.g., TMB (3,3′,5,5′-Tetramethylbenzidine) as redox species, the presence and quantity of biomarkers can be determined. This has been previously demonstrated by our group and is described elsewhere [14,20]. Thus, electrochemical detection on the microfluidic platform has the potential to enhance the field of detectable secreted proteins through high sensitivity sensing on a fully integrated platform, benefitting from enhanced mass transport as well as from redox amplification.

## Figures and Tables

**Table 1:** Stationary measurements in dual and single mode on platinum and carbon IDAs.

Parameter	Platinum IDA	Carbon IDA
Single mode peak current	5.2 μA	9.9 μA
Generator current	37.7 μA	165.5 μA
Collector current	37.5 μA	166.7 μA
Redox amplification (with respect to peak current)	7.25	16.7
Collector efficiency	0.99	1.0

**Table 2:** Single versus dual mode sensitivities and LOD for platinum IDA under flow.

Test Condition	Single Mode		Dual Mode	
Flow Rate (μL/min)	Sensitivity (μA/mM)	LOD (nM)	Sensitivity (μA/mM)	LOD (nM)
0.00	1.02	63.51	6.36	10.14
11.04	1.21	53.32	6.64	9.71
52.32	1.46	44.05	6.71	9.61
116.29	1.87	34.49	6.89	9.36
203.39	2.27	28.45	6.99	9.23

**Table 3:** Comparison of predicted and measured currents in dual mode at spin speed of 400 RPM at various concentrations.

Parameter	1 mM	3 mM	5 mM	7 mM	9 mM
Predicted Current (μA)	1.34	4.02	6.70	9.38	12.06
Measurement (μA)	5.62	19.43	37.67	52.38	60.95
